# Supplementary material for: Cryptic prokaryotic promoters explain instability of recombinant neuronal sodium channels in bacteria
Source: J Biol Chem. 2021 Jan 15;296:100298. doi: 10.1016/j.jbc.2021.100298 (PMC7948969; doi:10.1016/j.jbc.2021.100298)
Supplement: Figures S1 to S8 and Tables S1 and S2 [file mmc1.pdf]

Cryptic prokaryotic promoters explain instability of recombinant neuronal sodium channels in bacteria

Jean-Marc Dekeyser, Christopher H. Thompson, and Alfred L. George, Jr.

Supporting Information

Figure S1. Complete sequence of human $Na_V1.1$ cDNA with 60 stabilizing mutations

Figure S2. Partial human $Na_V1.1$ cDNA sequence (c.865-915) with a cryptic promoter

Figure S3. Nucleotide sequence of human HSPA5 intron 4

Figure S4. Gel images of stabilized human $Na_V1.1$, $Na_V1.2$, and $Na_V1.6$

Figure S5. Complete sequence of intron stabilized human $Na_V1.1$ cDNA

Figure S6. Complete sequence of intron stabilized human $Na_V1.2$ cDNA

Figure S7. Complete sequence of intron stabilized human $Na_V1.6$ cDNA

Figure S8. Electrophysiological properties of stabilized Na_V channels

Table S1 Predicted promoter-like elements in $Na_V1.1$ cassettes 1 and 2

Table S2 Mutations introduced to stabilize human $Na_V1.1$

Figure S1. Sequence of human $Na_V1.1$ with 60 synonymous stabilizing mutations shown as uppercase letters.

```
atggagcaaacagtgcttgtaccaccaggacctgaTagcttcaacttcttcaccagagaatctcttgcggctattga
aagacgcattgcagaagaaaaggcaaagaatcccaaaccagacaaaaaagatgacgacgaaaatggcccaaagccaa
atagtgacCtCgaagctggaaagaaccttccatttatttatggagacattcctccagagatggtgtcagagcccctg
gaggacctggacccctactatatcaataagaaaacttttatagtattgaataaagggaaggccatcttccggttcag
tgccacctctgccctgtacattCtCactcccttcaatcctcttaggaaaatagctattaagattttggtacattcat
tattcagcatgctaattatgtgcactattttgacaaactgtgtgtttatgacaatgagtaaccctcctgattggaca
aagaatgtagaatacaccttcacaggaatataCacCtttgaatcacttataaaaattatCgcaaggggattctgttt
agaagattttactttccttcgggaCccatggaactggctcgatttcactgtcattacatttgcgtacgtcacagagt
ttgtggacctgggcaatgtctcggcattgagaacattcagagttctccgagcattgaagacgatttcagtcattcca
ggcctgaaaaccattgtgggagccctgatccagtctgtgaagaagctctcagatgtaatgatcctgactgtgttctg
tctgagcgtatttgctctaattgggctgcagctgttcatgggcaacctgaggaataaatgtatTcaatggcctccca
ccaatgcttccCtCgaggaacatagtatagaGaagaatataactgtgaaCtaCaaCggtacacttataaatgaaact
gtcttCgagttCgactggaagtcataCatCcaagattcaagataCcaCtatttcctggagggtttttttagatgcact
actatgtggaaatagctctgatgcaggccaatgtccagagggatatatgtgtgtgaaagctggtagaaatcccaatt
atggctacacaagctttgataccttcagttgggctttttttgtccttgttCcgactaatgactcaggacttctgggaa
aaCctttatcaactgacattacgtgctgctgggaaaacgtacatgatattCttCgtattggtcattttcttgggctc
attctacctaataaatttgatcctggctgtCgtCgccatggcctacgaggaacagaaCcaggccaccttggaagaag
cagaacagaaagaggccgaatttcagcagatgattgaacagcttaaaaagcaacaggaggcagctcagcaggcagca
acggcaactgcctcagaacattccagagagcccagtgcagcaggcaggctctcagacagctcatctgaagcctctaa
gttgagttccaagagtgctaaggaaagaagaaatcggaggaagaaaagaaaacagaaagagcagtctggtggggaag
agaaagatgaggatgaattccaaaaatctgaatctgaggacagcatcaggaggaaaggttttcgcttctccattgaa
gggaaccgattgacatatgaaaagaggtactcctccccacaccagtctCtTtgagcatccgtggctccctattCtc
accaaggcgaaatagcagaacaagccttttcagctttagagggcgagcaaaggatgtgggatctgagaacgacttcg
cagatgatgagcacagcacctttgaggataacgagagccgtagagattccttgtttgtgccccgacgacacggagag
agacgcaacagcaacctgagtcagaccagtaggtcatcccggatgctggcagtgtttccagcgaatgggaagatgca
cagcactgtggattgcaatggtgtggtttccttggttggtggaccttcagttcctacatcgcctgtCggacagcttc
tgccagaggtgataatagataagccagctactgatgacaatggaacaaccactgaaactgaaatgagaaagagaagg
tcaagttctttccacgtttccatggactttctagaagatccttcccaaaggcaacgagcaatgagtatagccagcat
tctaacaaatacagtagaagaacttgaagaatccaggcagaaatgcccaccctgCtggtataaattttccaacatat
tcCtCatctgggactgttctccatattggttaaaagtgaaacatgttgtcaacctggttgtgatggacccattCgtC
gaTctggccatcaccatctgtattgtcCtaaaCactcttttcatggccatggagcactatccaatgacggaccattt
caataatgtgcttacagtaggaaacttggttttcactgggatcttCacCgcagaaatgtttctCaaaattattgcca
tggaCccttactattatttccaagaaggctggaatatcttCgacggttttattgtgacgcttagcctggtagaactt
ggactcgccaatgtggaaggattatctgttctccgttcatttcgaCtCctgcgagttttcaagCtCgcaaaatcCtg
gccaacgttaaatatgctaataaagatcatcggcaattccgtgggggctctgggaaatttaaccctcgtcttggcca
tcatcgtcttcattttCgccgtggtcggcatgcagctctttggtaaaagctacaaagattgtgtctgcaagatcgcc
agtgattgtcaactcccacgctggcacatgaatgacttcttccactccttcctgattgtgttccgcgtgctgtgtgg
ggagtggatagagaccatgtgggactgtatggaggttgctggtcaagccatgtgccttactgtcttcatgatggtca
tggtCattggaaacctagtggtcctgaatctcttCctggccttgcttctgagctcatttagtgcagacaacctCgca
gccactgatgatgataatgaaatgaaCaatctccaaattgctgtggaCaggatgcacaaaggagtcgcttatgtgaa
aagaaaaatatatgaGttCatCcaacagtccttcattaggaaacaaaagatttagatgaaattaaaccacttgatg
atctaaacaacaagaaagacagttgtatgtccaatcatacagcagaaattgggaaagatcttgactatcttaaagat
gtaaatggaactacaagtggtataggaactggcagcagtgttgaaaaatacattattgatgaaagtgattacatgtc
attcataaacaaccccagtcttactgtgactgtaccaattgctgtaggagaatctgactttgaaaatttaaacacgg
aagactttagtagtgaatcggatctggaagaaagcaaagagaaactgaatgaaagcagtagctcatcagaaggtagc
actgtggacatcggcgcacctgtagaagaacagcccgtagtggaacctgaagaaactcttgaaccagaagcttgttt
cactgaaggctgtgtacaaagattcaagtgttgtcaaatcaatgtggaagaaggcagaggaaaacaatggtggaacc
tgagaaggacgtgtttccgaatagttgaacataactggtttgagaccttcattgttttcatgattctccttagtagt
ggtgctctggcatttgaagatatatatattgatcagcgaaagacgattaagacgatgttggaatatgctgacaaggt
tttcacttacattttcattctggaaatgcttctaaaatgggtggcatatggctatcaaacatatttcaccaatgcct
ggtgttggctggacttcttaattgttgatgtttcattggtcagtttaacagcaaatgccttgggttactcagaactt
```

ggagccatcaaatctctcaggacactaagagctctgagacctctaagagccttatctcgatttgaagggatgagggt
ggttgtgaatgcccttttaggagcaattccatccatcatgaatgtgcttctggtttgtcttatattctggctaattt
tcagcatcatgggcgtaaatttgtttgctggcaaattctaccactgtattaacaccacaactggtgacaggttCgac
atcgaagacgtgaataatcatactgattgcctaaaactaatagaaagaaatgagactgctcgatggaaaaatgtgaa
agtaaactttgataatgtaggatttgggtatctctctttgcttcaagttgccacattcaaaggatggatggatataa
tgtatgcagcagttgattccagaaatgtggaactccagcctaagtatgaagaaagtctgtacatgtatctttacttt
gttattttcatcatctttgggtccttcttcaccttgaacctgtttattggtgtcatcatagataatttcaaccagca
gaaaaagaagtttggaggtcaagacatctttatgacagaagaacagaagaaatactataatgcaatgaaaaaattag
gatcgaaaaaaccgcaaaagcctatacctcgaccaggaaacaaatttcaaggaatggtctttgacttcgtaaccaga
caagtttttgacataagcatcatgattctcatctgtcttaacatggtcacaatgatggtggaaacagatgaccagag
tgaatatgtgactaccattttgtcacgcatcaatctggtgttcattgtgctatttactggagagtgtgtactgaaac
tcatctctctacgccattattattttaccattggatggaatatttttgattttgtggttgtcattctctccattgta
ggtatgtttcttgccgagctgatagaaaagtatttcgtgtcccctaccctgttccgagtgatccgtcttgctaggat
tggccgaatcctacgtctgatcaaaggagcaaaggggatccgcacgctgctctttgctttgatgatgtcccttcctg
cgttgtttaacatcggcctcctactcttcctagtcatgttcatctacgccatctttgggatgtccaactttgcctat
gttaagagggaagttgggatcgatgacatgttcaactttgagacctttggcaacagcatgatctgcctattccaaat
tacaacctctgctggctgggatggattgctagcacccattctcaacagtaagccacccgactgtgaccctaataaag
ttaaccctggaagctcagttaagggagactgtgggaacccatctgttggaatttttctttttttgtcagttacatcatc
atatccttcctggttgtggtgaacatgtacatcgcggtcatcctggagaacttcagtgttgctactgaagaaagtgc
agagcctctgagtgaggatgactttgagatgttctatgaggtttgggagaagtttgatcccgatgcaactcagttca
tggaatttgaaaaattatctcagtttgcagctgcgcttgaaccgcctctcaatctgccacaaccaaacaaactccag
ctcattgccatggatttgcccatggtgagtggtgaccggatccactgtcttgatatcttatttgcttttacaaagcg
ggttctaggagagagtggagagatggatgctctacgaatacagatggaagagcgattcatggcttccaatccttcca
aggtctcctatcagccaatcactactactttaaaacgaaaacaagaggaagtatctgctgtcattattcagcgtgct
tacagacgccaccttttaaagcgaactgtaaaacaagcttcctttacgtacaataaaaacaaaatcaaaggtggggc
taatcttcttataaaagaagacatgataattgacagaataaatgaaaactctattacagaaaaaactgatctgacca
tgtccactgcagcttgtccaccttcctatgaccgggtgacaaagccaattgtggaaaaacatgagcaagaaggcaaa
gatgaaaaagccaaagggaaataa

```
                      -35                    -10
                     TTGACANNNNNNNNNNNNNNNNNTATAAT
           E  H  S  I  E  K  N  I  T  V  N  Y  N  G  T  L  I
       865 gaacatagtaTAGAGAagaatataactgtgaatTATAATggtacacttata 915
    Stable ..............A.................C..C..C............
```

Figure S2. Partial human Na$_V$1.1 cDNA sequence (c.865-915) with a cryptic promoter. Partial amino acid and nucleic acid sequences of human Na$_V$1.1 cDNA highlighting promoter-like elements and the positions of engineered silent mutations made to the -35 or -10 boxes. Periods indicate wild type bases and letters indicate altered bases used to introduce silent mutations that inactivate the promoter. The -35 and -10 boxes are underlined and bolded. The consensus *E. coli* sigma 70 factor promoter sequence spanning Na$_V$1.1 c.875-903 is shown above.

```
          1                                                  50
WT Intron GTAAGTATGAAATTCAGGGATACGGCATATTTGCCAAATAGTGGAAATGT
v2 Intron ..................................................
          51                                    89
WT Intron GAAGTACTGACAAAACTTTTCCCTTTTTCAATCTAATAG
v2 Intron .............................CC...C....
```

Figure S3. Nucleotide sequence of human HSPA5 intron 4.

Canonical 5’ GT splice donor and 3’ AG splice acceptor sequences are underlined. Three nucleotide changes were made to generate the form (v2) used in the stable $Na_V1.6$ construct.

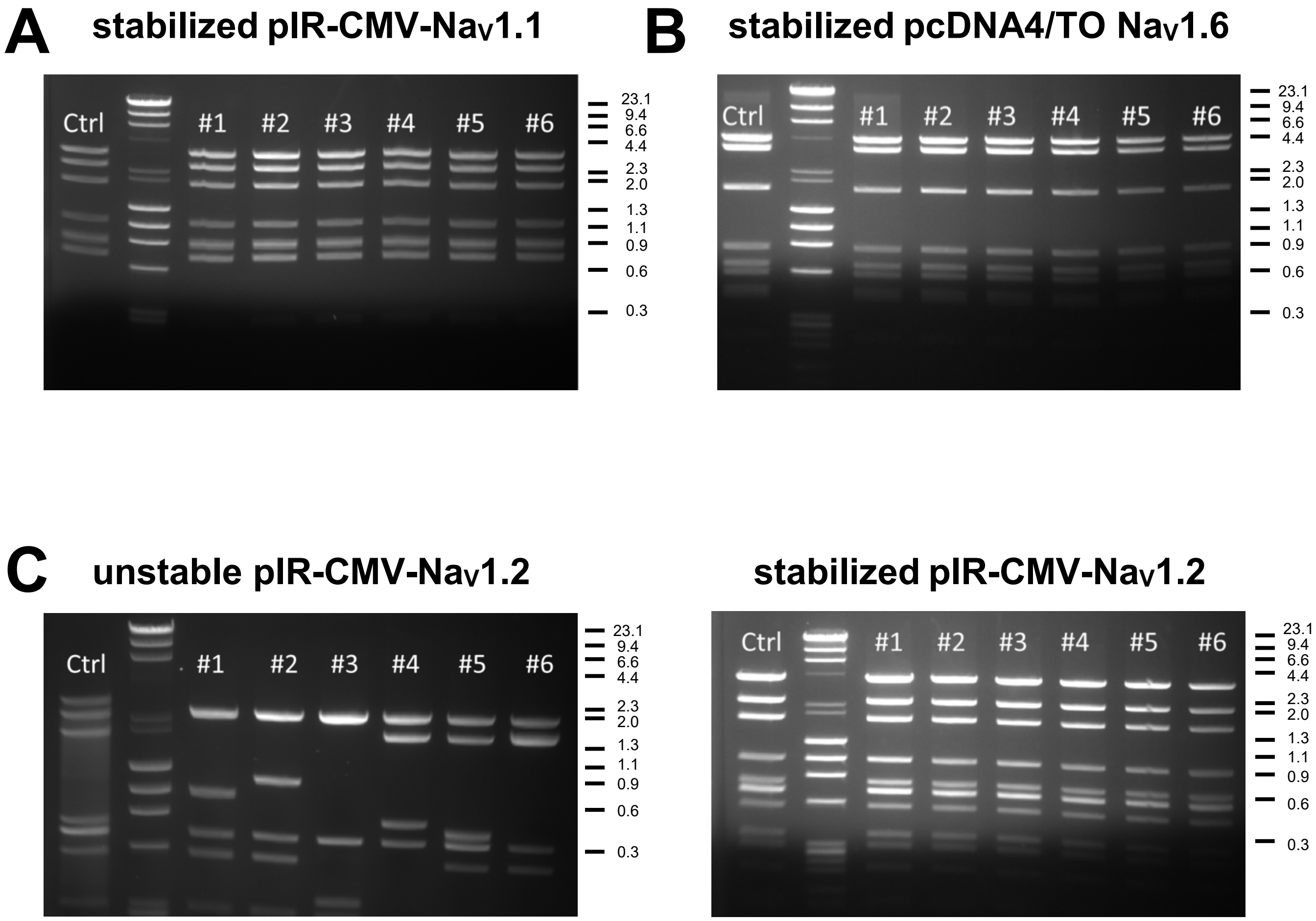

Figure S4. Gel images of stabilized human Na_V1.1, Na_V1.2, and Na_V1.6

Ethidium bromide stained *Nco*I restriction fragments resolved on 1% agarose gels for **A**) stabilized pIR-CMV-Na_V1.1; **B**) stabilized pcDNA4/TO-Na_V1.6; and **C**) unstable (left) and stabilized (right) pIR-CMV-Na_V1.2. Plasmids were isolated from overnight liquid cultures (LB-kanamycin, 50 µg/mL for Na_V1.1 and Na_V1.2, or LB-ampicillin, 100 µg/mL for Na_V1.6, 37ºC) inoculated with single colonies resulting from transformation of TOP10 cells and grown overnight at 37ºC. The restriction pattern of unstable Na_V plasmids isolated from Stbl2 cells are presented for comparison (left most lane, Ctrl). Unstable versions of pIR-CMV-Na_V1.1 and pcDNA4/TO-Na_V1.6 did not generate viable bacterial colonies following several attempts to transform TOP10 cells. The molecular weight marker is a mixture of *Hae*III digested bacteriophage ΦX174 DNA and *Hin*dIII digested bacteriophage λ DNA (between Ctrl and #1 lanes).

Figure S5. Sequence of intron stabilized human $Na_V1.1$ cDNA.

Changes made to the sequence of NM_001165963 included insertion of the IVS intron (blue lowercased text) inserted after c.2946; silent mutations (bold, underlined, yellow highlighted) c.879G>A, c.897T>C, c.900T>C and c.903T>C to disable the cryptic promoter in cassette 1 and at c.4155T>C to disable the cassette 3 cryptic promoter.

ATGGAGCAAACAGTGCTTGTACCACCAGGACCTGACAGCTTCAACTTCTTCACCAGAGAATCTCTTGCGGCTATTG
AAAGACGCATTGCAGAAGAAAAGGCAAAGAATCCCAAACCAGACAAAAAAGATGACGACGAAAATGGCCCAAAG
CCAAATAGTGACTTGGAAGCTGGAAAGAACCTTCCATTTATTTATGGAGACATTCCTCCAGAGATGGTGTCAGAGC
CCCTGGAGGACCTGGACCCCTACTATATCAATAAGAAAACTTTTATAGTATTGAATAAAGGGAAGGCCATCTTCCG
GTTCAGTGCCACCTCTGCCCTGTACATTTTAACTCCCTTCAATCCTCTTAGGAAAATAGCTATTAAGATTTTGGTACA
TTCATTATTCAGCATGCTAATTATGTGCACTATTTTGACAAACTGTGTGTTTATGACAATGAGTAACCCTCCTGATTG
GACAAAGAATGTAGAATACACCTTCACAGGAATATATACTTTTGAATCACTTATAAAAATTATTGCAAGGGGATTC
TGTTTAGAAGATTTTACTTTCCTTCGGGATCCATGGAACTGGCTCGATTTCACTGTCATTACATTTGCGTACGTCACA
GAGTTTGTGGACCTGGGCAATGTCTCGGCATTGAGAACATTCAGAGTTCTCCGAGCATTGAAGACGATTTCAGTCA
TTCCAGGCCTGAAAACCATTGTGGGAGCCCTGATCCAGTCTGTGAAGAAGCTCTCAGATGTAATGATCCTGACTGT
GTTCTGTCTGAGCGTATTTGCTCTAATTGGGCTGCAGCTGTTCATGGGCAACCTGAGGAATAAATGTATACAATGG
CCTCCCACCAATGCTTCCTTGGAGGAACATAGTATAGA**A**AAGAATATAACTGTGAA**C**TA**C**AA**C**GGTACACTTATAA
ATGAAACTGTCTTTGAGTTTGACTGGAAGTCATATATTCAAGATTCAAGATATCATTATTTCCTGGAGGGTTTTTA
GATGCACTACTATGTGGAAATAGCTCTGATGCAGGCCAATGTCCAGAGGGATATATGTGTGTGAAAGCTGGTAGA
AATCCCAATTATGGCTACACAAGCTTTGATACCTTCAGTTGGGCTTTTTTGTCCTTGTTTCGACTAATGACTCAGGAC
TTCTGGGAAAATCTTTATCAACTGACATTACGTGCTGCTGGGAAAACGTACATGATATTCTTCGTATTGGTCATTTT
CTTGGGCTCATTCTACCTAATAAATTTGATCCTGGCTGTGGTGGCCATGGCCTACGAGGAACAGAATCAGGCCACC
TTGGAAGAAGCAGAACAGAAAGAGGCCGAATTTCAGCAGATGATTGAACAGCTTAAAAAGCAACAGGAGGCAGC
TCAGCAGGCAGCAACGGCAACTGCCTCAGAACATTCCAGAGAGCCCAGTGCAGCAGGCAGGCTCTCAGACAGCTC
ATCTGAAGCCTCTAAGTTGAGTTCCAAGAGTGCTAAGGAAAGAAGAAATCGGAGGAAGAAAAGAAAACAGAAAG
AGCAGTCTGGTGGGGAAGAGAAAGATGAGGATGAATTCCAAAAATCTGAATCTGAGGACAGCATCAGGAGGAA
AGGTTTTCGCTTCTCCATTGAAGGGAACCGATTGACATATGAAAAGAGGTACTCCTCCCCACACCAGTCTTTGTTGA
GCATCCGTGGCTCCCTATTTTCACCAAGGCGAAATAGCAGAACAAGCCTTTTCAGCTTTAGAGGGCGAGCAAAGG
ATGTGGGATCTGAGAACGACTTCGCAGATGATGAGCACAGCACCTTTGAGGATAACGAGAGCCGTAGAGATTCCT
TGTTTGTGCCCCGACGACACGGAGAGAGACGCAACAGCAACCTGAGTCAGACCAGTAGGTCATCCCGGATGCTG
GCAGTGTTTCCAGCGAATGGGAAGATGCACAGCACTGTGGATTGCAATGGTGTGGTTTCCTTGGTTGGTGGACCT
TCAGTTCCTACATCGCCTGTTGGACAGCTTCTGCCAGAGGTGATAATAGATAAGCCAGCTACTGATGACAATGGAA
CAACCACTGAAACTGAAATGAGAAAGAGAAGGTCAAGTTCTTTCCACGTTTCCATGGACTTTCTAGAAGATCCTTC
CCAAAGGCAACGAGCAATGAGTATAGCCAGCATTCTAACAAATACAGTAGAAGAACTTGAAGAATCCAGGCAGA
AATGCCCACCCTGTTGGTATAAATTTTCCAACATATTCTTAATCTGGGACTGTTCTCCATATTGGTTAAAAGTGAAA
CATGTTGTCAACCTGGTTGTGATGGACCCATTTGTTGACCTGGCCATCACCATCTGTATTGTCTTAAATACTCTTTTC
ATGGCCATGGAGCACTATCCAATGACGGACCATTTCAATAATGTGCTTACAGTAGGAAACTTGGTTTTCACTGGGA
TCTTTACAGCAGAAATGTTTCTGAAAATTATTGCCATGGATCCTTACTATTATTTCCAAGAAGGCTGGAATATCTTT
GACGGTTTTATTGTGACGCTTAGCCTGGTAGAACTTGGACTCGCCAATGTGGAAGGATTATCTGTTCTCCGTTCATT
TCGATTGCTGCGAGTTTTCAAGTTGGCAAAATCTTGGCCAACGTTAAATATGCTAATAAAGATCATCGGCAATTCC
GTGGGGGCTCTGGGAAATTTAACCCTCGTCTTGGCCATCATCGTCTTCATTTTTGCCGTGGTCGGCATGCAGCTCTT
TGGTAAAAGCTACAAAGATTGTGTCTGCAAGATCGCCAGTGATTGTCAACTCCCACGCTGGCACATGAATGACTTC
TTCCACTCCTTCCTGATTGTGTTCCGCGTGCTGTGTGGGGAGTGGATAGAGACCATGTGGGACTGTATGGAGGTT

GCTGGTCAAGCCATGTGCCTTACTGTCTTCATGATGGTCATGGTGATTGGAAACCTAGTGgtaagtatcaaggttacaaga
caggtttaaggagaccaatagaaactgggcttgtcgagacagagaagactcttgcgtttctgataggcacctattggtcttactgacatccactttgcct
ttctctccacagGTCCTGAATCTCTTTCTGGCCTTGCTTCTGAGCTCATTTAGTGCAGACAACCTTGCAGCCACTGATGA
TGATAATGAAATGAATAATCTCCAAATTGCTGTGGATAGGATGCACAAAGGAGTAGCTTATGTGAAAAGAAAAAT
ATATGAATTTATTCAACAGTCCTTCATTAGGAAACAAAAGATTTTAGATGAAATTAAACCACTTGATGATCTAAACA
ACAAGAAAGACAGTTGTATGTCCAATCATACAGCAGAAATTGGGAAAGATCTTGACTATCTTAAAGATGTAAATG
GAACTACAAGTGGTATAGGAACTGGCAGCAGTGTTGAAAAATACATTATTGATGAAAGTGATTACATGTCATTCAT
AAACAACCCCAGTCTTACTGTGACTGTACCAATTGCTGTAGGAGAATCTGACTTTGAAAATTTAAACACGGAAGAC
TTTAGTAGTGAATCGGATCTGGAAGAAAGCAAAGAGAAACTGAATGAAAGCAGTAGCTCATCAGAAGGTAGCAC
TGTGGACATCGGCGCACCTGTAGAAGAACAGCCCGTAGTGGAACCTGAAGAAACTCTTGAACCAGAAGCTTGTTT
CACTGAAGGCTGTGTACAAAGATTCAAGTGTTGTCAAATCAATGTGGAAGAAGGCAGAGGAAAACAATGGTGGA
ACCTGAGAAGGACGTGTTTCCGAATAGTTGAACATAACTGGTTTGAGACCTTCATTGTTTTCATGATTCTCCTTAGT
AGTGGTGCTCTGGCATTTGAAGATATATATATTGATCAGCGAAAGACGATTAAGACGATGTTGGAATATGCTGAC
AAGGTTTTCACTTACATTTTCATTCTGGAAATGCTTCTAAAATGGGTGGCATATGGCTATCAAACATATTTCACCAA
TGCCTGGTGTTGGCTGGACTTCTTAATTGTTGATGTTTCATTGGTCAGTTTAACAGCAAATGCCTTGGGTTACTCAG
AACTTGGAGCCATCAAATCTCTCAGGACACTAAGAGCTCTGAGACCTCTAAGAGCCTTATCTCGATTTGAAGGGAT
GAGGGTGGTTGTGAATGCCCTTTTAGGAGCAATTCCATCCATCATGAATGTGCTTCTGGTTTGTCTTATATTCTGGC
TAATTTTCAGCATCATGGGCGTAAATTTGTTTGCTGGCAAATTCTACCACTGTATTAACACCACAACTGGTGACAGG
TT**C**GACATCGAAGACGTGAATAATCATACTGATTGCCTAAAACTAATAGAAAGAAATGAGACTGCTCGATGGAAA
AATGTGAAAGTAAACTTTGATAATGTAGGATTTGGGTATCTCTCTTTGCTTCAAGTTGCCACATTCAAAGGATGGAT
GGATATAATGTATGCAGCAGTTGATTCCAGAAATGTGGAACTCCAGCCTAAGTATGAAGAAAGTCTGTACATGTAT
CTTTACTTTGTTATTTTCATCATCTTTGGGTCCTTCTTCACCTTGAACCTGTTTATTGGTGTCATCATAGATAATTTCA
ACCAGCAGAAAAAGAAGTTTGGAGGTCAAGACATCTTTATGACAGAAGAACAGAAGAAATACTATAATGCAATGA
AAAAATTAGGATCGAAAAAACCGCAAAAGCCTATACCTCGACCAGGAAACAAATTTCAAGGAATGGTCTTTGACTT
CGTAACCAGACAAGTTTTTGACATAAGCATCATGATTCTCATCTGTCTTAACATGGTCACAATGATGGTGGAAACA
GATGACCAGAGTGAATATGTGACTACCATTTTGTCACGCATCAATCTGGTGTTCATTGTGCTATTTACTGGAGAGT
GTGTACTGAAACTCATCTCTCTACGCCATTATTATTTTACCATTGGATGGAATATTTTTGATTTTGTGGTTGTCATTCT
CTCCATTGTAGGTATGTTTCTTGCCGAGCTGATAGAAAAGTATTTCGTGTCCCCTACCCTGTTCCGAGTGATCCGTC
TTGCTAGGATTGGCCGAATCCTACGTCTGATCAAAGGAGCAAAGGGGATCCGCACGCTGCTCTTTGCTTTGATGAT
GTCCCTTCCTGCGTTGTTTAACATCGGCCTCCTACTCTTCCTAGTCATGTTCATCTACGCCATCTTTGGGATGTCCAA
CTTTGCCTATGTTAAGAGGGAAGTTGGGATCGATGACATGTTCAACTTTGAGACCTTTGGCAACAGCATGATCTGC
CTATTCCAAATTACAACCTCTGCTGGCTGGGATGGATTGCTAGCACCCATTCTCAACAGTAAGCCACCCGACTGTG
ACCCTAATAAAGTTAACCCTGGAAGCTCAGTTAAGGGAGACTGTGGGAACCCATCTGTTGGAATTTTCTTTTTTGTC
AGTTACATCATCATATCCTTCCTGGTTGTGGTGAACATGTACATCGCGGTCATCCTGGAGAACTTCAGTGTTGCTAC
TGAAGAAAGTGCAGAGCCTCTGAGTGAGGATGACTTTGAGATGTTCTATGAGGTTTGGGAGAAGTTTGATCCCGA
TGCAACTCAGTTCATGGAATTTGAAAAATTATCTCAGTTTGCAGCTGCGCTTGAACCGCCTCTCAATCTGCCACAAC
CAAACAAACTCCAGCTCATTGCCATGGATTTGCCCATGGTGAGTGGTGACCGGATCCACTGTCTTGATATCTTATTT
GCTTTTACAAAGCGGGTTCTAGGAGAGAGTGGAGAGATGGATGCTCTACGAATACAGATGGAAGAGCGATTCAT
GGCTTCCAATCCTTCCAAGGTCTCCTATCAGCCAATCACTACTACTTTAAAACGAAAACAAGAGGAAGTATCTGCTG
TCATTATTCAGCGTGCTTACAGACGCCACCTTTTAAAGCGAACTGTAAAACAAGCTTCCTTTACGTACAATAAAAAC
AAAATCAAAGGTGGGGCTAATCTTCTTATAAAAGAAGACATGATAATTGACAGAATAAATGAAAACTCTATTACAG
AAAAAACTGATCTGACCATGTCCACTGCAGCTTGTCCACCTTCCTATGACCGGGTGACAAAGCCAATTGTGGAAAA
ACATGAGCAAGAAGGCAAAGATGAAAAAGCCAAAGGGAAATAA

Figure S6. Sequence of intron stabilized human $Na_V1.2$ cDNA.
The only change made to the sequence of NM_021007 was insertion of the IVS intron (blue lower case text) after c.4551.

ATGGCACAGTCAGTGCTGGTACCGCCAGGACCTGACAGCTTCCGCTTCTTTACCAGGGAATCCCTTGCTGCTATTG
AACAACGCATTGCAGAAGAGAAAGCTAAGAGACCCAAACAGGAACGCAAGGATGAGGATGATGAAAATGGCCC
AAAGCCAAACAGTGACTTGGAAGCAGGAAAATCTCTTCCATTTATTTATGGAGACATTCCTCCAGAGATGGTGTCA
GTGCCCCTGGAGGATCTGGACCCCTACTATATCAATAAGAAAACGTTTATAGTATTGAATAAAGGGAAAGCAATCT
CTCGATTCAGTGCCACCCCTGCCCTTTACATTTTAACTCCCTTCAACCCTATTAGAAAATTAGCTATTAAGATTTTGG
TACATTCTTTATTCAATATGCTCATTATGTGCACGATTCTTACCAACTGTGTATTTATGACCATGAGTAACCCTCCAG
ACTGGACAAAGAATGTGGAGTATACCTTTACAGGAATTTATACTTTTGAATCACTTATTAAAATACTTGCAAGGGG
CTTTTGTTTAGAAGATTTCACATTTTTACGGGATCCATGGAATTGGTTGGATTTCACAGTCATTACTTTTGCATATGT
GACAGAGTTTGTGGACCTGGGCAATGTCTCAGCGTTGAGAACATTCAGAGTTCTCCGAGCATTGAAAACAATTTCA
GTCATTCCAGGCCTGAAGACCATTGTGGGGGCCCTGATCCAGTCAGTGAAGAAGCTTTCTGATGTCATGATCTTGA
CTGTGTTCTGTCTAAGCGTGTTTGCGCTAATAGGATTGCAGTTGTTCATGGGCAACCTACGAAATAAATGTTTGCA
ATGGCCTCCAGATAATTCTTCCTTTGAAATAAATATCACTTCCTTCTTTAACAATTCATTGGATGGGAATGGTACTAC
TTTCAATAGGACAGTGAGCATATTTAACTGGGATGAATATATTGAGGATAAAAGTCACTTTTATTTTTTAGAGGGG
CAAAATGATGCTCTGCTTTGTGGCAACAGCTCAGATGCAGGCCAGTGTCCTGAAGGATACATCTGTGTGAAGGCT
GGTAGAAACCCCAACTATGGCTACACGAGCTTTGACACCTTTAGTTGGGCCTTTTTGTCCTTATTTCGTCTCATGACT
CAAGACTTCTGGGAAAACCTTTATCAACTGACACTACGTGCTGCTGGGAAAACGTACATGATATTTTTTGTGCTGG
TCATTTTCTTGGGCTCATTCTATCTAATAAATTTGATCTTGGCTGTGGTGGCCATGGCCTATGAGGAACAGAATCAG
GCCACATTGGAAGAGGCTGAACAGAAGGAAGCTGAATTTCAGCAGATGCTCGAACAGTTGAAAAAGCAACAAGA
AGAAGCTCAGGCGGCAGCTGCAGCCGCATCTGCTGAATCAAGAGACTTCAGTGGTGCTGGTGGGATAGGAGTTTT
TTCAGAGAGTTCTTCAGTAGCATCTAAGTTGAGCTCCAAAAGTGAAAAAGAGCTGAAAAACAGAAGAAAGAAAAA
GAAACAGAAAGAACAGTCTGGAGAAGAAGAGAAAAATGACAGAGTCCGAAAATCGGAATCTGAAGACAGCATA
AGAAGAAAAGGTTTCCGTTTTTCCTTGGAAGGAAGTAGGCTGACATATGAAAAGAGATTTTCTTCTCCACACCAGT
CCTTACTGAGCATCCGTGGCTCCCTTTTCTCTCCAAGACGCAACAGTAGGGCGAGCCTTTTCAGCTTCAGAGGTCG
AGCAAAGGACATTGGCTCTGAGAATGACTTTGCTGATGATGAGCACAGCACCTTTGAGGACAATGACAGCCGAAG
AGACTCTCTGTTCGTGCCGCACAGACATGGAGAACGGCGCCACAGCAATGTCAGCCAGGCCAGCCGTGCCTCCAG
GGTGCTCCCCATCCTGCCCATGAATGGGAAGATGCATAGCGCTGTGGACTGCAATGGTGTGGTCTCCCTGGTCGG
GGGCCCTTCTACCCTCACATCTGCTGGGCAGCTCCTACCAGAGGGCACAACTACTGAAACAGAAATAAGAAAGAG
ACGGTCCAGTTCTTATCATGTTTCCATGGATTTATTGGAAGATCCTACATCAAGGCAAAGAGCAATGAGTATAGCC
AGTATTTTGACCAACACCATGGAAGAACTTGAAGAATCCAGACAGAAATGCCCACCATGCTGGTATAAATTTGCTA
ATATGTGTTTGATTTGGGACTGTTGTAAACCATGGTTAAAGGTGAAACACCTTGTCAACCTGGTTGTAATGGACCC
ATTTGTTGACCTGGCCATCACCATCTGCATTGTCTTAAATACACTCTTCATGGCTATGGAGCACTATCCCATGACGG
AGCAGTTCAGCAGTGTACTGTCTGTTGGAAACCTGGTCTTCACAGGGATCTTCACAGCAGAAATGTTTCTCAAGAT
AATTGCCATGGATCCATATTATTACTTTCAAGAAGGCTGGAATATTTTTGATGGTTTTATTGTGAGCCTTAGTTTAAT
GGAACTTGGTTTGGCAAATGTGGAAGGATTGTCAGTTCTCCGATCATTCCGGCTGCTCCGAGTTTTCAAGTTGGCA
AAATCTTGGCCAACTCTAAATATGCTAATTAAGATCATTGGCAATTCTGTGGGGGCTCTAGGAAACCTCACCTTGG
TATTGGCCATCATCGTCTTCATTTTTGCTGTGGTCGGCATGCAGCTCTTTGGTAAGAGCTACAAAGAATGTGTCTGC
AAGATTTCCAATGATTGTGAACTCCCACGCTGGCACATGCATGACTTTTTCCACTCCTTCCTGATCGTGTTCCGCGT
GCTGTGTGGAGAGTGGATAGAGACCATGTGGGACTGTATGGAGGTCGCTGGCCAAACCATGTGCCTTACTGTCTT
CATGATGGTCATGGTGATTGGAAATCTAGTGGTTCTGAACCTCTTCTTGGCCTTGCTTTTGAGTTCCTTCAGTTCTG
ACAATCTTGCTGCCACTGATGATGATAACGAAATGAATAATCTCCAGATTGCTGTGGGAAGGATGCAGAAAGGAA
TCGATTTTGTTAAAAGAAAAATACGTGAATTTATTCAGAAAGCCTTTGTTAGGAAGCAGAAAGCTTTAGATGAAAT

TAAACCGCTTGAAGATCTAAATAATAAAAAAGACAGCTGTATTTCCAACCATACCACCATAGAAATAGGCAAAGAC
CTCAATTATCTCAAAGACGGAAATGGAACTACTAGTGGCATAGGCAGCAGTGTAGAAAAATATGTCGTGGATGAA
AGTGATTACATGTCATTTATAAACAACCCTAGCCTCACTGTGACAGTACCAATTGCTGTTGGAGAATCTGACTTTGA
AAATTTAAATACTGAAGAATTCAGCAGCGAGTCAGATATGGAGGAAAGCAAAGAGAAGCTAAATGCAACTAGTTC
ATCTGAAGGCAGCACGGTTGATATTGGAGCTCCCGCCGAGGGAGAACAGCCTGAGGTTGAACCTGAGGAATCCC
TTGAACCTGAAGCCTGTTTTACAGAAGACTGTGTACGGAAGTTCAAGTGTTGTCAGATAAGCATAGAAGAAGGCA
AAGGGAAACTCTGGTGGAATTTGAGGAAAACATGCTATAAGATAGTGGAGCACAATTGGTTCGAAACCTTCATTG
TCTTCATGATTCTGCTGAGCAGTGGGGCTCTGGCCTTTGAAGATATATACATTGAGCAGCGAAAAACCATTAAGAC
CATGTTAGAATATGCTGACAAGGTTTTCACTTACATATTCATTCTGGAAATGCTGCTAAAGTGGGTTGCATATGGTT
TTCAAGTGTATTTTACCAATGCCTGGTGCTGGCTAGACTTCCTGATTGTTGATGTCTCACTGGTTAGCTTAACTGCA
AATGCCTTGGGTTACTCAGAACTTGGTGCCATCAAATCCCTCAGAACACTAAGAGCTCTGAGGCCACTGAGAGCTT
TGTCCCGGTTTGAAGGAATGAGGGTTGTTGTAAATGCTCTTTTAGGAGCCATTCCATCTATCATGAATGTACTTCTG
GTTTGTCTGATCTTTTGGCTAATATTCAGTATCATGGGAGTGAATCTCTTTGCTGGCAAGTTTTACCATTGTATTAAT
TACACCACTGGAGAGATGTTTGATGTAAGCGTGGTCAACAACTACAGTGAGTGCAAAGCTCTCATTGAGAGCAAT
CAAACTGCCAGGTGGAAAAATGTGAAAGTAAACTTTGATAACGTAGGACTTGGATATCTGTCTCTACTTCAAGTAG
CCACGTTTAAGGGATGGATGGATATTATGTATGCAGCTGTTGATTCACGAAATGTAGAATTACAACCCAAGTATGA
AGACAACCTGTACATGTATCTTTATTTTGTCATCTTTATTATTTTTGGTTCATTCTTTACCTTGAATCTTTTCATTGGTG
TCATCATAGATAACTTCAACCAACAGAAAAAGAAGTTTGGAGGTCAAGACATTTTTATGACAGAAGAACAGAAGA
AATACTACAATGCAATGAAAAAACTGGGTTCAAAGAAACCACAAAAACCCATACCTCGACCTGCTgtaagtatcaaggt
tacaagacaggtttaaggagaccaatagaaactgggcttgtcgagacagagaagactcttgcgtttctgataggcacctattggtcttactgacatcca
ctttgcctttctctccacagAACAAATTCCAAGGAATGGTCTTTGATTTTGTAACCAAACAAGTCTTTGATATCAGCATCAT
GATCCTCATCTGCCTTAACATGGTCACCATGATGGTGGAAACCGATGACCAGAGTCAAGAAATGACAAACATTCTG
TACTGGATTAATCTGGTGTTTATTGTTCTGTTCACTGGAGAATGTGTGCTGAAACTGATCTCTCTTCGTTACTACTAT
TTCACTATTGGATGGAATATTTTTGATTTTGTGGTGGTCATTCTCTCCATTGTAGGAATGTTTCTGGCTGAACTGATA
GAAAAGTATTTTGTGTCCCCTACCCTGTTCCGAGTGATCCGTCTTGCCAGGATTGGCCGAATCCTACGTCTGATCAA
AGGAGCAAAGGGGATCCGCACGCTGCTCTTTGCTTTGATGATGTCCCTTCCTGCGTTGTTTAACATCGGCCTCCTTC
TTTTCCTGGTCATGTTCATCTACGCCATCTTTGGGATGTCCAATTTTGCCTATGTTAAGAGGGAAGTTGGGATCGAT
GACATGTTCAACTTTGAGACCTTTGGCAACAGCATGATCTGCCTGTTCCAAATTACAACCTCTGCTGGCTGGGATG
GATTGCTAGCACCTATTCTTAATAGTGGACCTCCAGACTGTGACCCTGACAAAGATCACCCTGGAAGCTCAGTTAA
AGGAGACTGTGGGAACCCATCTGTTGGGATTTTCTTTTTTGTCAGTTACATCATCATATCCTTCCTGGTTGTGGTGA
ACATGTACATCGCGGTCATCCTGGAGAACTTCAGTGTTGCTACTGAAGAAAGTGCAGAGCCTCTGAGTGAGGATG
ACTTTGAGATGTTCTATGAGGTTTGGGAGAAGTTTGATCCCGATGCGACCCAGTTTATAGAGTTTGCCAAACTTTCT
GATTTTGCAGATGCCCTGGATCCTCCTCTTCTCATAGCAAAACCCAACAAAGTCCAGCTCATTGCCATGGATCTGCC
CATGGTGAGTGGTGACCGGATCCACTGTCTTGACATCTTATTTGCTTTTACAAAGCGTGTTTTGGGTGAGAGTGGA
GAGATGGATGCCCTTCGAATACAGATGGAAGAGCGATTCATGGCATCAAACCCCTCCAAAGTCTCTTATGAGCCCA
TTACGACCACGTTGAAACGCAAACAAGAGGAGGTGTCTGCTATTATTATCCAGAGGGCTTACAGACGCTACCTCTT
GAAGCAAAAAGTTAAAAAGGTATCAAGTATATACAAGAAAGACAAAGGCAAAGAATGTGATGGAACACCCATCA
AAGAAGATACTCTCATTGATAAACTGAATGAGAATTCAACTCCAGAGAAAACCGATATGACGCCTTCCACCACGTC
TCCACCCTCGTATGATAGTGTGACCAAACCAGAAAAAGAAAAATTTGAAAAAGACAAATCAGAAAAGGAAGACAA
AGGGAAAGATATCAGGGAAAGTAAAAAGTAA

Figure S7. Sequence of intron stabilized human $Na_V1.6$ cDNA.

Changes made to NM_014191 included insertion of modified HSPA5 intron 4 (red lower case text) inserted after c.2370, insertion of IVS intron (blue lower case text) inserted after c.4281, silent mutations (bold, underlined, yellow highlighted) c.4119A>G and c.4122T>C to disable a cryptic promoter.

ATGGCAGCGCGGCTGCTTGCACCACCAGGCCCTGATAGTTTCAAGCCTTTCACCCCTGAGTCACTGGCAAACATTG
AGAGGCGCATTGCTGAGAGCAAGCTCAAGAAACCACCAAAGGCCGATGGCAGTCATCGGGAGGACGATGAGGA
CAGCAAGCCCAAGCCAAACAGCGACCTGGAAGCAGGGAAGAGTTTGCCTTTCATCTACGGGGACATCCCCCAAGG
CCTGGTTGCAGTTCCCCTGGAGGACTTTGACCCATACTATTTGACGCAGAAAACCTTTGTAGTATTAAACAGAGGG
AAAACTCTCTTCAGATTTAGTGCCACGCCTGCCTTGTACATTTTAAGTCCTTTTAACCTGATAAGAAGAATAGCTATT
AAAATTTTGATACATTCAGTATTTAGCATGATCATTATGTGCACTATTTTGACCAACTGTGTATTCATGACTTTTAGT
AACCCTCCTGACTGGTCGAAGAATGTGGAGTACACGTTCACAGGGATTTATACATTTGAATCACTAGTGAAAATCA
TTGCAAGAGGTTTCTGCATAGATGGCTTTACCTTTTTACGGGACCCATGGAACTGGTTAGATTTCAGTGTCATCATG
ATGGCGTATATAACAGAGTTTGTAAACCTAGGCAATGTTTCAGCTCTACGCACTTTCAGGGTACTGAGGGCTTTGA
AAACTATTTCGGTAATCCCAGGCCTGAAGACAATTGTGGGTGCCCTGATTCAGTCTGTGAAGAAACTGTCAGATGT
GATGATCCTGACAGTGTTCTGCCTGAGTGTTTTTGCCTTGATCGGACTGCAGCTGTTCATGGGGAACCTTCGAAAC
AAGTGTGTTGTGTGGCCCATAAACTTCAACGAGAGCTATCTTGAAAATGGCACCAAAGGCTTTGATTGGGAAGAG
TATATCAACAATAAAACAAATTTCTACACAGTTCCTGGCATGCTGGAACCTTTACTCTGTGGGAACAGTTCTGATGC
TGGGCAATGCCCAGAGGGATACCAGTGTATGAAAGCAGGAAGGAACCCCAACTATGGTTACACAAGTTTTGACAC
TTTTAGCTGGGCCTTCTTGGCATTATTTCGCCTTATGACCCAGGACTATTGGGAAAACTTGTATCAATTGACTTTAC
GAGCAGCCGGGAAAACATACATGATCTTCTTCGTCTTGGTCATCTTTGTGGGTTCTTTCTATCTGGTGAACTTGATC
TTGGCTGTGGTGGCCATGGCTTATGAAGAACAGAATCAGGCAACACTGGAGGAGGCAGAACAAAAAGAGGCTGA
ATTTAAAGCAATGTTGGAGCAACTTAAGAAGCAACAGGAAGAGGCACAGGCTGCTGCGATGGCCACTTCAGCAG
GAACTGTCTCAGAAGATGCCATAGAGGAAGAAGGTGAAGAAGGAGGGGGCTCCCCTCGGAGCTCTTCTGAAATC
TCTAAACTCAGCTCAAAGAGTGCAAAGGAAAGACGTAACAGGAGAAAGAAGAGGAAGCAAAAGGAACTCTCTGA
AGGAGAGGAGAAAGGGGATCCCGAGAAGGTGTTTAAGTCAGAGTCAGAAGATGGCATGAGAAGGAAGGCCTTT
CGGCTGCCAGACAACAGAATAGGGAGGAAATTTTCCATCATGAATCAGTCACTGCTCAGCATCCCAGGCTCGCCCT
TCCTCTCCCGCCACAACAGCAAGAGCAGCATCTTCAGTTTCAGGGGACCTGGGCGGTTCCGAGACCCGGGCTCCG
AGAATGAGTTCGCGGATGACGAGCACAGCACGGTGGAGGAGAGCGAGGGCCGCCGGGACTCCCTCTTCATCCCC
ATCCGGGCCCGCGAGCGCCGGAGCAGCTACAGCGGCTACAGCGGCTACAGCCAGGGCAGCCGCTCCTCGCGCAT
CTTCCCCAGCCTGCGGCGCAGCGTGAAGCGCAACAGCACGGTGGACTGCAACGGCGTGGTGTCCCTCATCGGCG
GCCCCGGCTCCCACATCGGCGGGCGTCTCCTGCCAGAGGCTACAACTGAGGTGGAAATTAAGAAGAAAGGCCCT
GGATCTCTTTTAGTTTCCATGGACCAATTAGCCTCCTACGGGCGGAAGGACAGAATCAACAGTATAATGAGTGTTG
TTACAAATACACTAGTAGAAGAACTGGAAGAGTCTCAGAGAAAGTGCCCGCCATGCTGGTATAAATTTGCCAACA
CTTTCCTCATCTGGGAGTGCCACCCCTACTGGATAAAACTGAAAGAGATTGTGAACTTGATAGTTATGGACCCTTTT
GTGGATTTAGCCATCACCATCTGCATCGTCCTGAATACACTGTTTATGGCAATGGAGCACCATCCTATGACACCACA
ATTTGAACATGTCTTGGCTGTAGGAAATCTGgtaagtatgaaattcagggatacggcatatttgccaaatagtggaaatgtgaagtact
gacaaaacttttccctttttccctctcatagGTTTTCACTGGAATTTTCACAGCGGAAATGTTCCTGAAGCTCATAGCCATGGAT
CCCTACTATTATTTCCAAGAAGGTTGGAACATTTTTGACGGATTTATTGTCTCCCTCAGTTTAATGGAACTGAGTCTA
GCAGACGTGGAGGGGCTTTCAGTGCTGCGATCTTTCCGATTGCTCCGAGTCTTCAAATTGGCCAAATCCTGGCCCA
CCCTGAACATGCTAATCAAGATTATTGGAAATTCAGTGGGTGCCCTGGGCAACCTGACACTGGTGCTGGCCATTAT
TGTCTTCATCTTTGCCGTGGTGGGGATGCAACTCTTTGGAAAAAGCTACAAAGAGTGTGTCTGCAAGATCAACCAG
GACTGTGAACTCCCTCGCTGGCATATGCATGACTTTTTCCATTCCTTCCTCATTGTCTTTCGAGTGTTGTGCGGGGA
GTGGATTGAGACCATGTGGGACTGCATGGAAGTGGCAGGCCAGGCCATGTGCCTCATTGTCTTTATGATGGTCAT

GGTGATTGGCAACTTGGTGGTGCTGAACCTGTTTCTGGCCTTGCTCCTGAGCTCCTTCAGTGCAGACAACCTGGCT
GCCACAGATGACGATGGGGAAATGAACAACCTCCAGATCTCAGTGATCCGTATCAAGAAGGGTGTGGCCTGGACC
AAACTAAAGGTGCACGCCTTCATGCAGGCCCACTTTAAGCAGCGTGAGGCTGATGAGGTGAAGCCTCTGGATGAG
TTGTATGAAAAGAAGGCCAACTGTATCGCCAATCACACCGGTGCAGACATCCACCGGAATGGTGACTTCCAGAAG
AATGGCAATGGCACAACCAGCGGCATTGGCAGCAGCGTGGAGAAGTACATCATTGATGAGGACCACATGTCCTTC
ATCAACAACCCCAACTTGACTGTACGGGTACCCATTGCTGTGGGCGAGTCTGACTTTGAGAACCTCAACACAGAGG
ATGTTAGCAGCGAGTCGGATCCTGAAGGCAGCAAAGATAAACTAGATGACACCAGCTCCTCTGAAGGAAGCACCA
TTGATATCAAACCAGAAGTAGAAGAGGTCCCTGTGGAACAGCCTGAGGAATACTTGGATCCAGATGCCTGCTTCA
CAGAAGGTTGTGTCCAGCGGTTCAAGTGCTGCCAGGTCAACATCGAGGAAGGGCTAGGCAAGTCTTGGTGGATC
CTGCGGAAAACCTGCTTCCTCATCGTGGAGCACAACTGGTTTGAGACCTTCATCATCTTCATGATTCTGCTGAGCAG
TGGCGCCCTGGCCTTCGAGGACATCTACATTGAGCAGAGAAAGACCATCCGCACCATCCTGGAATATGCTGACAA
AGTCTTCACCTATATCTTCATCCTGGAGATGTTGCTCAAGTGGACAGCCTATGGCTTCGTCAAGTTCTTCACCAATG
CCTGGTGTTGGCTGGACTTCCTCATTGTGGCTGTCTCTTTAGTCAGCCTTATAGCTAATGCCCTGGGCTACTCGGAA
CTAGGTGCCATAAAGTCCCTTAGGACCCTAAGAGCTTTGAGACCCTTAAGAGCCTTATCACGATTTGAAGGGATGA
GGGTGGTGGTGAATGCCTTGGTGGGCGCCATCCCCTCCATCATGAATGTGCTGCTGGTGTGTCTCATCTTCTGGCT
GATTTTCAGCATCATGGGAGTTAACTTGTTTGCGGGAAAGTACCACTACTGCTTTAATGAGACTTCTGAAATCCGAT
TTGAAATTGAAGATGTCAACAATAA**G**AC**C**GAATGTGAAAAGCTTATGGAGGGGAACAATACAGAGATCAGATGG
AAGAACGTGAAGATCAACTTTGACAATGTTGGGGCAGGATACCTGGCCCTTCTTCAAGTAGCAACCTTCAAAGGCT
GGATGGACATCATGTATGCAGCTGTAGATTCCCGGAAGgtaagtatcaaggttacaagacaggtttaaggagaccaatagaaact
gggcttgtcgagacagagaagactcttgcgtttctgataggcacctattggtcttactgacatccactttgcctttctctccacagCCTGATGAGCA
GCCTAAGTATGAGGACAATATCTACATGTACATCTATTTTGTCATCTTCATCATCTTCGGCTCCTTCTTCACCCTGAA
CCTGTTCATTGGTGTCATCATTGATAACTTCAATCAACAAAAGAAAAAGTTCGGAGGTCAGGACATCTTCATGACC
GAAGAACAGAAGAAGTACTACAATGCCATGAAAAAGCTGGGCTCAAAGAAGCCACAGAAACCTATTCCCCGCCCC
TTGAACAAAATCCAAGGAATCGTCTTTGATTTTGTCACTCAGCAAGCCTTTGACATTGTTATCATGATGCTCATCTG
CCTTAACATGGTGACAATGATGGTGGAGACAGACACTCAAAGCAAGCAGATGGAGAACATCCTCTACTGGATTAA
CCTGGTGTTTGTTATCTTCTTCACCTGTGAGTGTGTGCTCAAAATGTTTGCGTTGAGGCACTACTACTTCACCATTG
GCTGGAACATCTTCGACTTCGTGGTAGTCATCCTCTCCATTGTGGGAATGTTCCTGGCAGATATAATTGAGAAATA
CTTTGTTTCCCCAACCCTATTCCGAGTCATCCGATTGGCCCGTATTGGGCGCATCTTGCGTCTGATCAAAGGCGCCA
AAGGGATTCGTACCCTGCTCTTTGCCTTAATGATGTCCTTGCCTGCCCTGTTCAACATCGGCCTTCTGCTCTTCCTGG
TCATGTTCATCTTCTCCATTTTTGGGATGTCCAATTTTGCATATGTGAAGCACGAGGCTGGTATCGATGACATGTTC
AACTTTGAGACATTTGGCAACAGCATGATCTGCCTGTTTCAAATCACAACCTCAGCTGGTTGGGATGGCCTGCTGC
TGCCCATCCTAAACCGCCCCCCTGACTGCAGCCTAGATAAGGAACACCCAGGGAGTGGCTTTAAGGGAGATTGTG
GGAACCCCTCAGTGGGCATCTTCTTCTTTGTAAGCTACATCATCATCTCTTTCCTAATTGTCGTGAACATGTACATTG
CCATCATCCTGGAGAACTTCAGTGTAGCCACAGAGGAAAGTGCAGACCCTCTGAGTGAGGATGACTTTGAGACCT
TCTATGAGATCTGGGAGAAGTTCGACCCCGATGCCACCCAGTTCATTGAGTACTGTAAGCTGGCAGACTTTGCAGA
TGCCTTGGAGCATCCTCTCCGAGTGCCCAAGCCCAATACCATTGAGCTCATCGCTATGGATCTGCCAATGGTGAGC
GGGGATCGCATCCACTGCTTGGACATCCTTTTTGCCTTCACCAAGCGGGTCCTGGGAGATAGCGGGGAGTTGGAC
ATCCTGCGGCAGCAGATGGAAGAGCGGTTCGTGGCATCCAATCCTTCCAAAGTGTCTTACGAGCCAATCACAACC
ACACTGCGTCGCAAGCAGGAGGAGGTATCTGCAGTGGTCCTGCAGCGTGCCTACCGGGGACATTTGGCAAGGCG
GGGCTTCATCTGCAAAAAGACAACTTCTAATAAGCTGGAGAATGGAGGCACACACCGGGAGAAAAAGAGAGCA
CCCCATCTACAGCCTCCCTCCCGTCCTATGACAGTGTAACTAAACCTGAAAAGGAGAAACAGCAGCGGGCAGAGG
AAGGAAGAAGGGAAAGAGCCAAAAGACAAAAAGAGGTCAGAGAATCCAAGTGTTGA

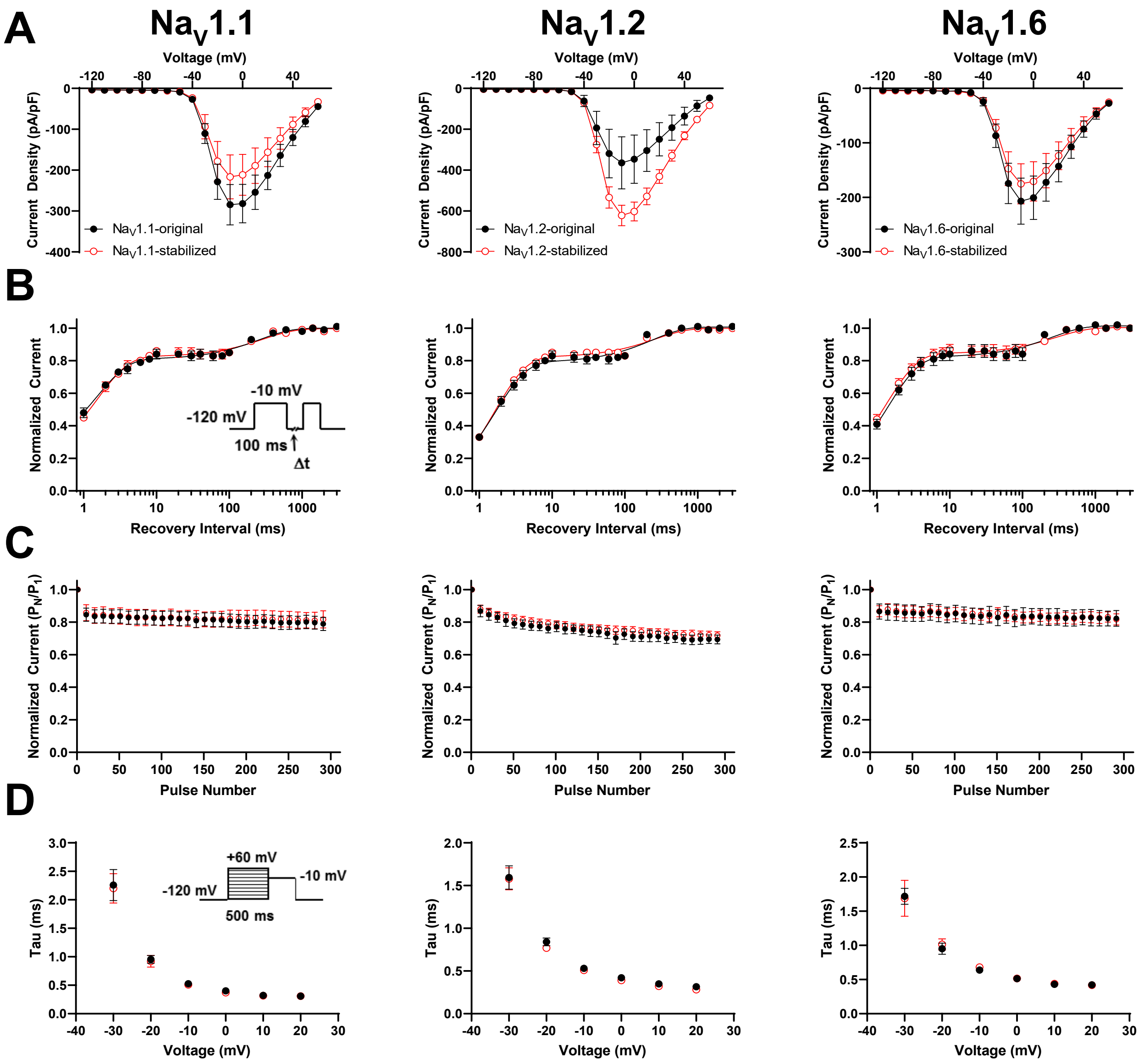

Figure S8. Electrophysiological properties of stabilized NaV channels.

Additional electrophysiological data for each channel are arranged vertically beneath each heading in the top panel. **A**) Current-voltage relationships recorded from cells expressing either unstable (black symbols and lines) or stabilized (red symbols and lines) $Na_V1.1$, $Na_V1.2$ and $Na_V1.6$. Currents were normalized to cell capacitance to calculate current density and plotted as mean ± SEM. **B**) Time course of recovery from inactivation was measured following a 100 ms step depolarization to -10 mV followed by a variable length recovery step to -120 mV and a test depolarization (4 ms, -10 mV). Lines were generated by average fits of the data with a two-exponential function. **C**) Frequency dependent rundown assessed by 300 depolarizing pulses (4 ms) to 0 mV elicited at a frequency of 50 Hz. **D**). Voltage-dependence of fast inactivation time constant derived from exponential fits of the whole-cell recordings illustrated in Fig. 5B.

Table S1 – Predicted promoter-like elements in $Na_V1.1$ cassettes 1 and 2.

Start	End	Score[1]	Sequence
27	72	0.86	AGGACCTGACAGCTTCAACTTCTTCACCAGAGAATCTCTTGCGGCTATTG
158	203	0.95	GTGACTTGGAAGCTGGAAAGAACCTTCCATTTATTTATGGAGACATTCCT
325	370	0.99	TACATTTTAACTCCCTTCAATCCTCTTAGGAAAATAGCTATTAAGATTTT
348	393	0.83	TCTTAGGAAAATAGCTATTAAGATTTTGGTACATTCATTATTCAGCATGC
367	412	0.84	AAGATTTTGGTACATTCATTATTCAGCATGCTAATTATGTGCACTATTTT
464	509	0.95	AGAATGTAGAATACACCTTCACAGGAATATATACTTTTGAATCACTTATA
515	560	0.92	AAATTATTGCAAGGGGATTCTGTTTAGAAGATTTTACTTTCCTTCGGGAT
856	901	0.92	TCCTTGGAGGAACATAGTATAGAAAAGAATATAACTGTGAACTACAACGG
**869**	**914**	**0.93**	**ATAGTATAGAAAAGAATATAACTGTGAATTATAATGGTACACTTATAAAT**
**869**	**914**	**0.86**	**ATAGTATAGAAAAGAATATAACTGTGAA<u>C</u>TA<u>C</u>AA<u>C</u>GGTACACTTATAAAT**[2]
933	978	0.92	GTTTGACTGGAAGTCATATATTCAAGATTCAAGATATCATTATTTCCTGG
972	1017	0.82	TTATTTCCTGGAGGGTTTTTTAGATGCACTACTATGTGGAAATAGCTCTG
1124	1169	0.94	TGTTTCGACTAATGACTCAGGACTTCTGGGAAAATCTTTATCAACTGACA
1984	2029	0.97	TCGCCTGTTGGACAGCTTCTGCCAGAGGTGATAATAGATAAGCCAGCTAC
2302	2347	0.99	CCATTTGTTGACCTGGCCATCACCATCTGTATTGTCTTAAATACTCTTTT
2425	2470	0.89	GGGATCTTTACAGCAGAAATGTTTCTGAAAATTATTGCCATGGATCCTTA
2444	2489	0.95	TGTTTCTGAAAATTATTGCCATGGATCCTTACTATTATTTCCAAGAAGGC
2580	2625	0.93	ATTTCGATTGCTGCGAGTTTTCAAGTTGGCAAAATCTTGGCCAACGTTAA
2602	2647	0.97	AAGTTGGCAAAATCTTGGCCAACGTTAAATATGCTAATAAAGATCATCGG
2614	2659	0.86	TCTTGGCCAACGTTAAATATGCTAATAAAGATCATCGGCAATTCCGTGGG
2926	2971	0.98	ATGGTGATTGGAAACCTAGTGGTCCTGAATCTCTTTCTGGCCTTGCTTCT
2992	3037	0.81	GACAACCTTGCAGCCACTGATGATGATAATGAAATGAATAATCTCCAAAT
3022	3067	0.8	GAAATGAATAATCTCCAAATTGCTGTGGATAGGATGCACAAAGGAGTAGC
3039	3084	0.95	AATTGCTGTGGATAGGATGCACAAAGGAGTAGCTTATGTGAAAAGAAAAA
3068	3113	0.89	TAGCTTATGTGAAAAGAAAAATATATGAATTTATTCAACAGTCCTTCATT

[1]from Berkeley Drosophila Genome Project Neural Network Promoter Prediction (https://fruitfly.org/seq_tools/promoter.html)

[2]Promoter from 869-914 (bold) was identified as an active promoter and was mutated (red, underlined)

Table S2 – Mutations introduced to stabilize human $Na_V1.1$ in the low promoter construct

c.C36T	c.T969C	c.A2436C
c.T163C	c.T972C	c.G2451C
c.G165C	c.T1128C	c.T2469C
c.T331C	c.T1158C	c.T2505C
c.A333C	c.G1263C	c.T2587C
c.T495C	c.G1266C	c.G2589C
c.T498C	c.T1290C	c.T2605C
c.T522C	c.T1666C	c.G2607C
c.T564C	c.G1668T	c.T2616A
c.A834T	c.T1692C	c.T2712C
c.T859C	c.T1992C	c.G2931C
c.G861C	c.T2211C	c.T2961C
c.A879G	c.T2236C	c.T3000C
c.T897C	c.A2238C	c.T3030C
c.T900C	c.T2307C	c.T3051C
c.T903C	c.T2310C	c.A3069C
c.T930C	c.C2313T	c.A3096G
c.T936C	c.T2338C	c.T3099C
c.T951C	c.T2343C	c.T3102C
c.T954C	c.T2433C	c.T4155C
